# Office‐Based Blue Laser Therapy for Vocal Fold Leukoplakia: A Preliminary Report of 12 Cases

**DOI:** 10.1002/oto2.59

**Published:** 2023-06-15

**Authors:** Abdul‐Latif Hamdan, Anthony Ghanem, Patrick Abou Raji Feghali, Jad Hosri, Christophe Abi Zeid Daou, Samer Abou‐Rizk

**Affiliations:** ^1^ Department of Otolaryngology and Head & Neck Surgery American University of Beirut Medical Center Beirut Lebanon; ^2^ Department of Otolaryngology and Head & Neck Surgery Lebanese American University Medical Center‐Rizk Hospital Beirut Lebanon

**Keywords:** blue laser, dysphonia, office‐based, vocal fold leukoplakia

## Abstract

**Objective:**

To report the efficacy of office‐based blue laser therapy for vocal fold leukoplakia.

**Study Design:**

A retrospective case series.

**Setting:**

A tertiary care center.

**Methods:**

A retrospective chart review of patients with vocal fold leukoplakia who underwent office‐based blue laser therapy between July 2019 and October 2022 was conducted. The video recordings of their laryngeal examination and their voice evaluation were analyzed before and after surgical intervention.

**Results:**

A total of 10 patients, eight with unilateral disease and 2 with bilateral disease, were included in this study. In total, 12 vocal folds with leukoplakia were treated. Nine had a single session and 3 had 2 sessions due to incomplete regression of the lesion after the first laser therapy session. Following treatment, 9 regressed completely (75%) and 3 regressed partially (25%). The mean Voice Handicap Index‐10 (VHI‐10) score decreased significantly from 15.4 ± 12.9 preoperatively to 3.8 ± 2.86 after surgery (*p* = .023). There was a statistically significant decrease in the means of grade, roughness, breathiness, asthenia, and strain (*p* < .05). There was also a statistically significant decrease in the jitter and shimmer percent (*p* = .008 and *p* = .048, respectively) and a significant increase in the maximum phonation time from 9.63 ± 3.83 to 13.54 ± 5.92 seconds (*p* = .039).

**Conclusion:**

This preliminary study indicates that office‐based blue laser therapy is an effective treatment modality for vocal fold leukoplakia.

Leukoplakia is defined as a whitish discoloration of the mucosal lining.^1^ Its occurrence in the upper aerodigestive system has been associated with several risk factors most important of which are smoking, laryngopharyngeal reflux disease, and genetic predisposition.^2^, ^3^ When the vocal fold is affected, leukoplakia invariably denotes a pathologic transformation of the underlying mucosal cover. The pathologic changes can range from epithelial hyperplasia to dysplasia and carcinoma. The prevalence of dysplasia varies with an average occurrence of 50%.^4^, ^5^, ^6^ Its presence is usually alarming given the unpredictable course of the disease and the high rate of malignant transformation. In a review of 940 cases, Weller et al reported malignant transformation in 14% within a mean follow‐up period of 5.8 years.^7^

Several treatment modalities for vocal fold leukoplakia have been suggested. These include vocal hygiene therapy with behavioral modification,^8^ frequent biopsies/serial excision,^9^ and laser ablation. In 2015, Zhang et al compared the efficacy of carbon dioxide (CO_2_) laser versus conventional laryngeal microsurgery in the treatment of vocal fold polyps and leukoplakia. Using subjective (Grade, Roughness, Breathiness, Asthenia, and Strain [GRBAS], Voice Handicap Index‐10 [VHI‐10]) and objective voice measures (acoustic spectrum analysis), the authors showed a better recovery in the laser group compared to the conventional group, especially in patients with vocal fold leukoplakia.^10^ Similarly, in 2018, Lim et al reported the use of the CO_2_ laser and angiolytic lasers (potassium titanyl phosphate [KTP] and pulse dye laser [PDL]) in the treatment of vocal fold leukoplakia under general anesthesia. Only 1 out of 5 had disease recurrence and the angiolytic laser‐stripping group had better voice preservation compared to the CO_2_ laser treatment group.^11^

With the advances in technology, laser therapy for vocal fold leukoplakia is now being performed in office. The most used lasers are the photoangiolytic lasers, namely the PDL with a wavelength of 583 nm and the KTP laser with a wavelength of 532 nm.^12^, ^13^, ^14^ Recently, a new laser with a wavelength of 445 nm (blue laser) has been approved by the Food and Drug Administration and is commonly used as a substitute for the KTP laser. Its hybrid properties, cutting, and coagulation, have increased its versatility in the treatment of different laryngeal pathology. The application of blue laser in the management of vocal fold leukoplakia has been reported only in isolated cases.^15^, ^16^, ^17^ Miller et al reported their experience with the blue laser in 29 patients, 4 of whom had suspicious lesions for which laser‐assisted diagnostic biopsy was performed. The authors did not elaborate on the pathology of these lesions.^16^ Hamdan et al in their series of 11 patients who underwent office‐based blue laser therapy described one case of vocal fold leukoplakia that regressed completely following a single treatment session, and another case of carcinoma in situ, which needed radiation therapy.^17^

Given the scarcity of reports on office‐based blue laser therapy, the authors of this manuscript report their experience with the use of blue laser in 12 cases of vocal fold leukoplakia treated in‐office.

## Materials and Methods

This study was conducted according to the Declaration of Helsinki and obtained approval from the Institutional Review Board of the American University of Beirut Medical Center (IRB ID: BIO‐2022‐0280). A retrospective chart review of patients with vocal fold leukoplakia who underwent office‐based biopsy followed by blue laser therapy between July 2019 and October 2022 was conducted. The patients included in this review have not been previously reported or included in any prior publication.

Demographic data included age, gender, smoking history, laterality of the lesion (right vs left), size (less than or greater than half the vocal fold), and pathology. A comprehensive voice evaluation was performed before and after surgical intervention. It comprised the VHI‐10 described by Rosen et al,^18^ GRBAS grading described by Hirano et al,^19^ and acoustic analysis using the VISI‐PITCH IV software (model 3950B; Kay Pentax). The maximum phonation time (MPT) was also reported as an aerodynamic measure. The acoustic analysis included the fundamental frequency (*F*_0_), habitual pitch (HP), percent shimmer, percent jitter, noise‐to‐harmonic ratio (NHR), and voice turbulence index (VTI). The extent of disease regression seen on laryngeal endoscopy was assessed before and after surgery over a follow‐up period that extended from 3 to 14 months. Laryngeal video‐stroboscopic examinations were also analyzed when available looking at glottic closure and malleability of the vocal fold cover.

The video recordings were reviewed by 2 otolaryngologists independently who rated disease regression following laser therapy. The inter‐rater variability was computed to assess the reliability of the reported findings.

### Statistical Method

Categorical and continuous variables were described using frequencies and means (±standard deviation), respectively. Using the Statistical Package for the Social Sciences (SPSS) version 24 software package, paired *t* test was used to analyze continuous variables before and after the intervention. A 2‐tailed *p* < .05 was considered statistically significant.

### Surgical Technique

**Figure 1:**
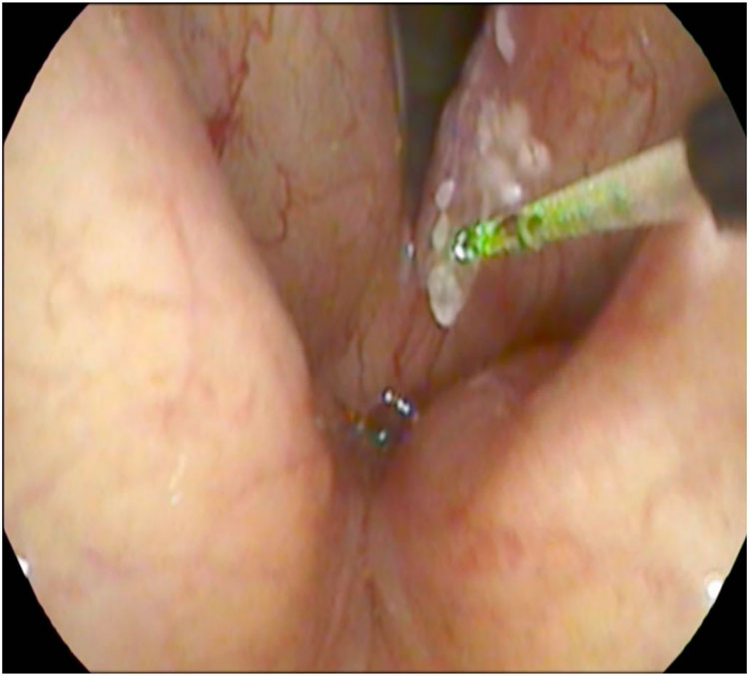
An endoscopic view of the larynx showing the blue laser glass fiber (400 μm) in near contact mode during the treatment of a left vocal fold leukoplakia.

**Figure 2:**
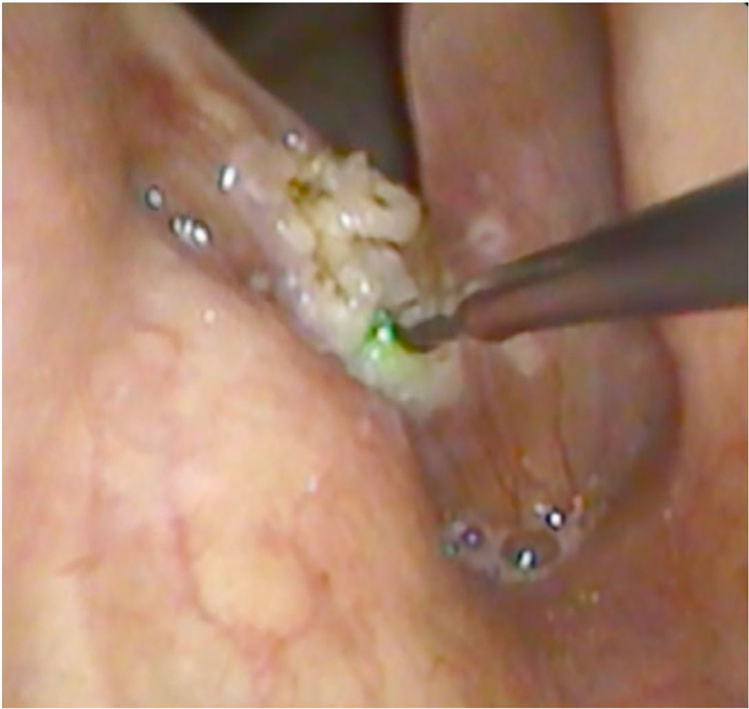
An endoscopic view of the larynx showing the blue laser glass fiber (400 μm) in contact mode during the treatment of a right vocal fold leukoplakia.

With the patient fully awake and in the upright sitting position, sponges soaked with 1% lidocaine hydrochloride (HCL) and 1:100,000 Epinephrine were inserted and kept in both nasal cavities for 10 minutes to achieve local anesthesia and decongestion. Xylocaine spray (2%) was used (2‐3 puffs) to anesthetize the oropharynx and hypopharynx. Laryngeal anesthesia was achieved using the “laryngeal gargle” technique described by Hogikyan.^20^ The flexible nasopharyngoscope (Pentax Medical FNL‐15RP3) with a working channel was introduced through the nasal cavity to the oropharynx and hypopharynx, and 2 ccs of 4% lidocaine were dripped through the working channel of the endoscope while the patient was asked to sustain the vowel /eh/. Following the application of local anesthesia to the laryngopharyngeal complex, a 400‐μm glass fiber was introduced through the working channel and directed toward the site of the lesion. The blue laser (TrueBlue; A.R.C. Laser Company) was administered in the pulse mode using the following set‐up: 10 W power, 30 ms pulse duration, and 300 ms interpulse duration. The laser was used in the near‐contact and contact modes (see Figures 1 and 2). The tip of the fiber was used at times to curette the surgical debris after laser therapy. All procedures were well‐tolerated by the patients.

## Results

### Demographic Data

A total of 10 patients, eight with unilateral disease and 2 with bilateral disease were included in this study. This study group has not been reviewed or included in previous studies. In total, 12 vocal folds with leukoplakia were treated. The mean age of the study group was 64.8 ± 10.3 years. The male‐to‐female ratio was 7:3. All patients included in this study were smokers. The majority of the lesions occupied less than half the vocal folds (n = 8) and the most common pathology was low‐grade dysplasia (n = 5). All patients had a single procedure except for 2 patients who underwent 2 procedures each (see Table 1).

**Table 1:** Demographic Characteristics of the Study Population

Case	Age	Gender	Smoker	Laterality	Size of lesion	Final pathology	Last follow‐up
1	70	Male	Yes	Left	<½ of vocal fold	Hyperkeratosis	12 months
2	81	Male	Yes	Right	<½ of vocal fold	High‐grade dysplasia	12 months
3	75	Male	Yes	Right	<½ of vocal fold	Low‐grade dysplasia	12 months
4	74	Female	Yes	Right	>½ of vocal fold	High‐grade dysplasia	13 months
5	57	Female	Yes	Right	>½ of vocal fold	Hyperkeratosis	14 months
6	57	Male	No	Right	<½ of vocal fold	Low‐grade dysplasia	8 months
7	53	Male	Yes	Right	<½ of vocal fold	Hyperkeratosis	6 months
8	61	Female	Yes	Left	<½ of vocal fold	High‐grade dysplasia	3 months
9	51	Male	Yes	Left	<½ of vocal fold	Low‐grade dysplasia	5 months
				Right	<½ of vocal fold	Low‐grade dysplasia	
10	70	Male	Yes	Left	Entire vocal fold	High‐grade dysplasia	5 months
				Right	Entire vocal fold	Low‐grade dysplasia	

### Disease Regression After Office‐Based Blue Laser Therapy

**Figure 3:**
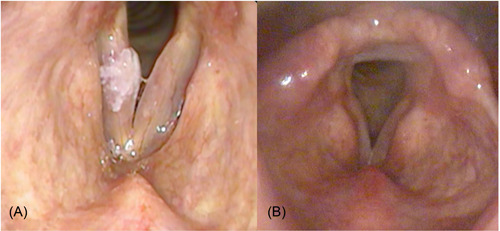
An endoscopic view of the larynx showing a right vocal fold leukoplakia before treatment (A) and after (B). Note the complete regression of the lesion following therapy.

**Figure 4:**
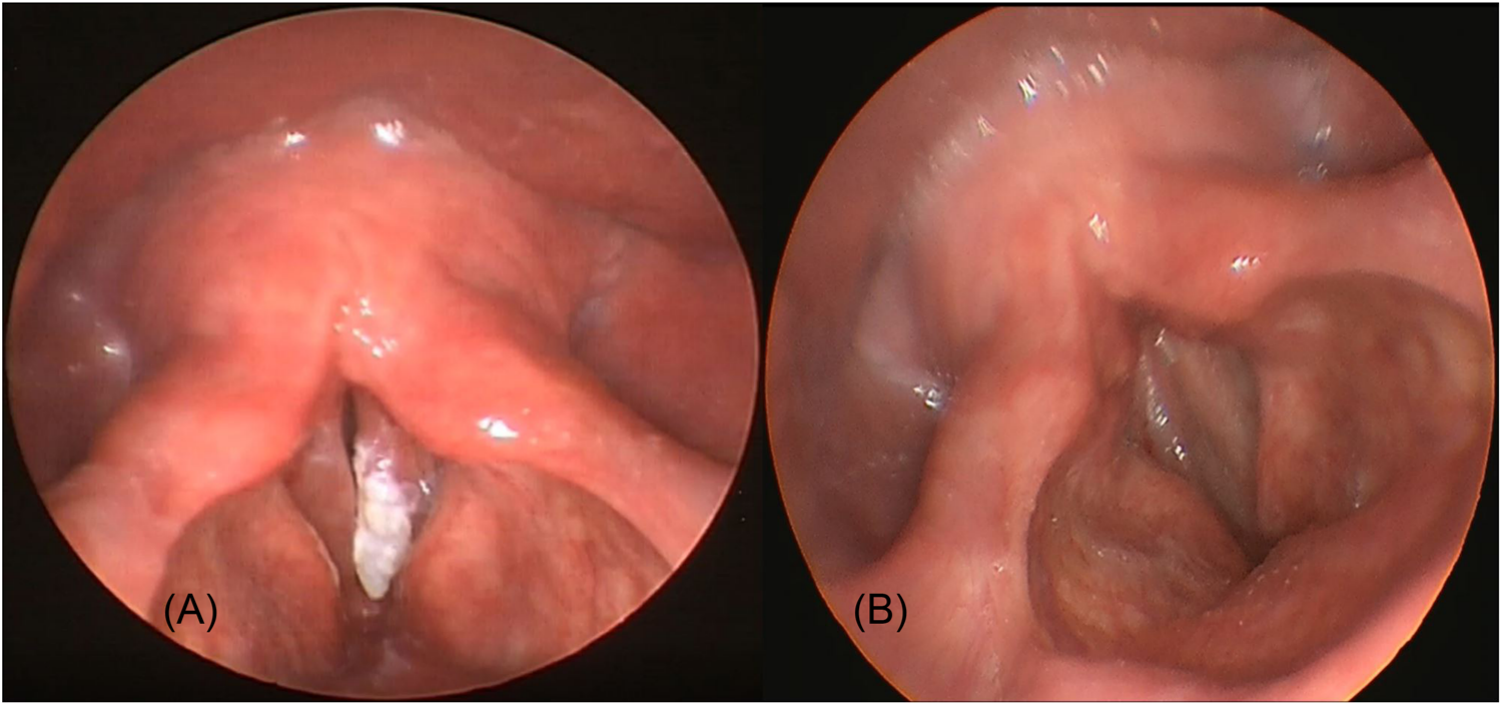
An endoscopic view of the larynx showing leukoplakia of left vocal fold (A). Note total regression of the lesion and complete closure of the vocal folds during phonation on video‐stroboscopic examination (B).

All patients tolerated well office‐based blue laser surgery with no complications noted. The average duration of surgery was 7.16 ± 2.84 minutes with a range from 1.87 to 13.07 minutes. The average amount of joules delivered during surgery which was noted in 5 of the 10 patients was 147.2 ± 88.2 J. The mean follow‐up period of the study group was 9 ± 4.03 months, with a range of 3 to 14 months. Out of the 12 vocal fold lesions treated, eight regressed completely (67%) and 4 regressed partially (33%) after a single session of laser therapy. Of those 4 lesions, 3 were treated with another session of office‐based blue laser therapy following which there was complete regression in 1 and partial regression in 2. In total, after completion of office‐based laser therapy, 9 of the 12 regressed completely (75%) and 3 regressed partially (25%) (see Figure 3). Laryngeal video‐stroboscopic examinations pre‐ and postsurgery were available in 7 patients. A review of these video recordings showed improvement in the mucosal waves and better glottic closure after surgery in all patients (see Figure 4).

Inter‐rater reliability analysis revealed an intraclass correlation coefficient (ICC) of 0.837 indicating excellent reliability between the 2 otolaryngologists who evaluated the endoscopic laryngeal findings.

### VHI‐10 and GRBAS Scores Before and After Laser Therapy

The mean VHI‐10 score of the total group decreased significantly from 15.4 ± 12.9 preoperatively to 3.8 ± 2.86 after surgery (*p* = .023). Eight of the 10 patients had a drop in their VHI‐10 score postoperatively, while the remaining 2 had no change. There was also a statistically significant decrease in the means of grade, roughness, breathiness, asthenia, and strain from 2.56 ± 0.73 to 0.78 ± 0.45 (*p* < .001), 2.56 ± 0.78 to 0.78 ± 0.45 (*p* < .001), 1.0 ± 0.87 to 0.33 ± 0.5 (*p* = .022), 0.78 ± 0.44 to 0.22 ± 0.44 (*p* = .013), and from 0.89 ± 0.6 to 0.22 ± 0.44 (*p* = .004) (see Table 2).

**Table 2:** Mean ± Standard Deviation of Voice Handicap Index and GRBAS Scores Before and After Office‐Based Blue Laser Treatment

	Before	After	*p* Value
VHI‐10	15.4 ± 12.9	3.8 ± 2.86	.023*
GRBAS			
Grade	2.56 ± 0.73	0.78 ± 0.45	<.001*
Roughness	2.56 ± 0.73	0.78 ± 0.45	<.001*
Breathiness	1.0 ± 0.87	0.33 ± 0.50	.022*
Asthenia	0.78 ± 0.44	0.22 ± 0.44	.013*
Strain	0.89 ± 0.60	0.22 ± 0.44	.004*

Abbreviations: GRBAS, Grade, Roughness, Breathiness, Asthenia, and Strain; VHI, Voice Handicap Index;

* Statistically significant (*p* < .05).

### Acoustic and Aerodynamic Analysis Before and After Surgery

There was a statistically significant decrease in the jitter percent from 1.27 ± 0.52 preoperatively to 0.72 ± 0.16 postoperatively (*p* = .008), and in the shimmer percent value from 4.13 ± 1.84 to 2.55 ± 0.89 (*p* = .048). The decrease in NHR from 0.135 ± 0.03 to 0.12 ± 0.02 and the decrease in VTI from 0.048 ± 0.018 to 0.039 ± 0.009 were not significant (*p* = .091 and *p* = .299, respectively) (see Table 3). The mean fundamental frequencies and habitual pitch of the study group before and after surgery, stratified by gender are displayed in Table 4.

**Table 3:** Means ± Standard Deviation of Acoustic Analysis Parameters and Maximum Phonation Time Before and After Blue Laser Treatment

	Before	After	*p* Value
Jitter (%)	1.27 ± 0.52	0.72 ± 0.16	.008*
Shimmer (%)	4.13 ± 1.84	2.55 ± 0.89	.048*
NHR	0.135 ± 0.03	0.12 ± 0.015	.091
VTI	0.049 ± 0.02	0.039 ± 0.009	.299
MPT (s)	9.63 ± 3.83	13.54 ± 5.92	.039*

Abbreviations: MPT, maximum phonation time; NHR, noise‐to‐harmonic ratio; VTI, Voice Turbulence Index.

* Statistically significant (*p* < .05).

**Table 4:** Mean Scores of Fundamental Frequency (*F*_0_) Hz and Habitual Pitch Before and After Blue Laser Therapy

	Male		Female	
	Fundamental frequency	Habitual pitch	Fundamental frequency	Habitual pitch
Before	119.34 ± 28.8	121.12 ± 23.01	175.53 ± 54.6	172.3 ± 58.7
After	111.76 ± 24.8	107.91 ± 15.37	180.47 ± 55.5	166.8 ± 51.5
*p* Value	.463	.226	.095	.389

With respect to the aerodynamic analysis, there was a significant increase in the MPT from 9.63 ± 3.83 to 13.54 ± 5.92 seconds (*p* = .039).

## Discussion

The last 2 decades witnessed a shift in laryngeal surgery from the operating room to the office. This shift is ascribed mostly to the introduction of the flexible endoscope with a working channel and fiber‐based lasers. The ability to deliver laser via a glass fiber has allowed laryngologists to treat benign and per‐malignant lesions of the vocal folds under local anesthesia. There are numerous reports in the literature on the use of photoangiolytic lasers in the management of vocal fold leukoplakia/dysplasia, mostly using the PDL or KTP laser. In 2006, Zeitels et al reported the use of KTP laser for the treatment of 36 patients with vocal fold dysplasia in an office setting and reported 75% disease regression in 62% of the cases. In the remaining patients, there was at least 25% disease regression.^14^ In 2007, Koufman et al reviewed 443 cases of office‐based laser surgery that included 79 cases of glottal dysplasia. The authors reported partial or complete responses in the majority of the cases using different types of lasers.^12^ In 2007, Mouadeb and Belafsky reported their experience with PDL in 47 patients, 4 of whom had vocal fold leukoplakia. Two patients were successfully treated in the office and 2 required a visit to the operating room.^13^ In 2017, Koss et al reported serial in‐office laser treatment of vocal fold leukoplakia using the KTP or PDL lasers in 46 patients who underwent a median of 2 (range: 1‐6) procedures. The authors noted complete disease regression in 41.3% and partial disease regression in 54.4% of the patients. Twenty‐eight percent of those who had partial disease regression underwent further surgical intervention in the operating room, 2 of whom were followed by irradiation.^21^ In 2017, Hu et al evaluated the feasibility and limitations of office‐based CO_2_ laser surgery for a variety of laryngeal lesions that included 13 cases of vocal fold leukoplakia. Of the 11 cases who underwent surgery, 9 had complete remission, while 2 had disease recurrence in the subglottic region of the larynx.^22^

There is no case series on the use of blue laser for the treatment of vocal fold leukoplakia in an office setting. The results of our study are in alignment with previous reports using other types of lasers. Three out of 4 patients in our study group had complete disease regression and one‐third had partial disease regression following office‐based blue laser therapy. The disease regression was associated with improvement in both subjective and objective voice outcome measures. There was a significant decrease in the VHI‐10 and GRBAS scores, and a significant decrease in the jitter and shimmer percent after treatment. There was also a significant increase in MPT commensurate with improvement in glottic closure during phonation seen in the majority of the cases. The response to treatment can be ascribed to the great affinity of the blue laser for oxyhemoglobin, which allows angiolysis of the sublesional microvasculature thus leading to disease regression.^23^ Using electron microscopic examination, Zeitels et al noted denaturation of the basement membrane with separation of the epithelial lining and destruction of the intraepithelial desmosome junctions following KTP laser therapy.^23^ These changes were primarily ascribed to selective photo‐angiolysis of the subepithelial microvasculature.

This study has its limitations. One is its retrospective nature, which allows for the inherent bias in patient selection as most of the lesions treated (8 of 12) did not exceed half the vocal fold. Patients with bigger lesions would probably have less favorable outcomes than those with smaller lesions as a result of less tolerance as reported by Zheng et al.^24^ Second is the relatively small number of subjects which may underpower this study. Another limitation is the short follow‐up period in some patients which does not allow us to evaluate the recurrence of disease.

## Conclusion

This is the first case series on office‐based blue laser therapy for vocal fold with leukoplakia. The results of this preliminary report indicate that blue laser therapy leads to disease regression and improvement in subjective and objective voice measures in most cases. In‐office blue laser therapy may be considered as an effective treatment modality with diligent observation for the need for more than 1 laser session in some cases.

## Author Contributions

**Abdul‐Latif Hamdan**, design and writing of the manuscript; **Anthony Ghanem**, design and writing of the manuscript; **Patrick Abou Raji Feghali**, collection and analysis of data; **Jad Hosri**, collection and analysis of data; **Christophe Abi Zeid Daou**, critical review and final version approval; **Samer Abou Rizk**, critical review and final version approval.

## Disclosures

### Competing interests

The authors declare that there is no conflict of interest.

### Funding

None.

## Data Availability

The data that support the findings of this study are available from the corresponding author, upon reasonable request.

## References

[1] Bouquot JE , Gnepp DR . Laryngeal precancer: a review of the literature, commentary, and comparison with oral leukoplakia. Head Neck. 1991;13(6):488‐497. 10.1002/hed.2880130604 1791144

[2] Marcos CÁ , Alonso‐Guervós M , Prado NR , et al. Genetic model of transformation and neoplastic progression in laryngeal epithelium. Head Neck. 2011;33(2):216‐224. 10.1002/hed.21432 20629083

[3] Zhou J , Zhang D , Yang Y , Zhou L , Tao L . Association between helicobacter pylori infection and carcinoma of the larynx or pharynx. Head Neck. 2016;38(suppl 1):E2291‐E2296. 10.1002/hed.24214 26316145

[4] Isenberg JS , Crozier DL , Dailey SH . Institutional and comprehensive review of laryngeal leukoplakia. Ann Otol Rhinol Laryngol. 2008;117(1):74‐79. 10.1177/000348940811700114 18254375

[5] Cui W , Xu W , Yang Q , Hu R . Clinicopathological parameters associated with histological background and recurrence after surgical intervention of vocal cord leukoplakia. Medicine. 2017;96(22):e7033. 10.1097/MD.0000000000007033 28562558 PMC5459723

[6] Gale N , Michaels L , Luzar B , et al. Current review on squamous intraepithelial lesions of the larynx. Histopathology. 2009;54(6):639‐656. 10.1111/j.1365-2559.2008.03111.x 18752537

[7] Weller MD , Nankivell PC , McConkey C , Paleri V , Mehanna HM . The risk and interval to malignancy of patients with laryngeal dysplasia; a systematic review of case series and meta‐analysis. Clin Otolaryngol. 2010;35(5):364‐372. 10.1111/j.1749-4486.2010.02181.x 21108746

[8] Gao XW , Huang YW , Liu LY , Ouyang J . Use Videostrobokymography to quantitatively analyze the vibratory characteristics before and after conservative medical treatment of vocal fold leukoplakia. J Voice. 2016;30(2):215‐220. 10.1016/j.jvoice.2015.04.015 26001502

[9] Schweinfurth JM , Powitzky E , Ossoff RH . Regression of laryngeal dysplasia after serial microflap excision. Ann Otol Rhinol Laryngol. 2001;110(9):811‐814. 10.1177/000348940111000902 11558755

[10] Zhang Y , Liang G , Sun N , et al. Comparison of CO2 laser and conventional laryngomicrosurgery treatments of polyp and leukoplakia of the vocal fold. Int J Clin Exp Med. 2015;8(10):18265‐18274. 26770428 PMC4694328

[11] Lim JY , Park YM , Kang M , et al. Angiolytic laser stripping versus CO2 laser microflap excision for vocal fold leukoplakia: long‐term disease control and voice outcomes. PLoS One. 2018;13(12):e0209691. 10.1371/journal.pone.0209691 30596718 PMC6312374

[12] Koufman JA , Rees CJ , Frazier WD , et al. Office‐based laryngeal laser surgery: a review of 443 cases using three wavelengths. Otolaryngol Head Neck Surg. 2007;137(1):146‐151. 10.1016/j.otohns.2007.02.041 17599582

[13] Mouadeb DA , Belafsky PC . In‐office laryngeal surgery with the 585nm pulsed dye laser (PDL). Otolaryngol Head Neck Surg. 2007;137(3):477‐481. 10.1016/j.otohns.2007.02.003 17765779

[14] Zeitels SM , Akst LM , Burns JA , Hillman RE , Broadhurst MS , Anderson RR . Office‐based 532‐nm pulsed KTP laser treatment of glottal papillomatosis and dysplasia. Ann Otol Rhinol Laryngol. 2006;115(9):679‐685. 10.1177/000348940611500905 17044539

[15] Hess MM , Fleischer S , Ernstberger M . New 445 nm blue laser for laryngeal surgery combines photoangiolytic and cutting properties. Eur Arch Otrhinolaryngol. 2018;275(6):1557‐1567. 10.1007/s00405-018-4974-8 29675755

[16] Miller BJ , Abdelhamid A , Karagama Y . Applications of office‐based 445 nm blue laser transnasal flexible laser surgery: a case series and review of practice. Ear Nose Throat J. 2021;100(1_suppl):105S‐112S. 10.1177/0145561320960544 32970490

[17] Hamdan AL , Ghanem A . Un‐sedated office‐based application of blue laser in vocal fold lesions. J Voice. 2021. 10.1016/j.jvoice.2021.03.031 34030923

[18] Rosen CA , Lee AS , Osborne J , Zullo T , Murry T . Development and validation of the voice handicap index‐10. Laryngoscope. 2004;114(9):1549‐1556. 10.1097/00005537-200409000-00009 15475780

[19] Hirano M . GRBAS” scale for evaluating the hoarse voice & frequency range of phonation. In: Wyke BD , ed. Clinical Examination of Voice. Vol 5, 1981:83‐84.

[20] Hogikyan ND . How I do it head and neck and plastic surgery: a targeted problem and its solution: transnasal endoscopic examination of the subglottis and trachea using topical anesthesia in the otolaryngology clinic. Laryngoscope. 1999;109(7 pt 1):1170‐1173. 10.1097/00005537-199907000-00032 10401864

[21] Koss SL , Baxter P , Panossian H , Woo P , Pitman MJ . Serial in‐office laser treatment of vocal fold leukoplakia: Disease control and voice outcomes. Laryngoscope. 2017;127(7):1644‐1651. 10.1002/lary.26445 28083976

[22] Hu HC , Lin SY , Hung YT , Chang SY . Feasibility and associated limitations of office‐based laryngeal surgery using carbon dioxide lasers. JAMA Otolaryngol Head Neck Surg. 2017;143(5):485‐491. 10.1001/jamaoto.2016.4129 28208177 PMC5824306

[23] Zeitels SM , Burns JA , Lopez‐Guerra G , Anderson RR , Hillman RE . Photoangiolytic laser treatment of early glottic cancer: a new management strategy. Ann Otol Rhinol Laryngol Suppl. 2008;117:3‐24. 10.1177/00034894081170s701 18710131

[24] Zheng M , Arora N , Bhatt N , O'Dell K , Johns, 3rd M . Factors associated with tolerance for in‐office laryngeal laser procedures. Laryngoscope. 2021;131(7):E2292‐E2297. 10.1002/lary.29370 33405311

